# Multimodal Imaging of Retinal Changes in a Patient Taking Axitinib

**DOI:** 10.2174/0115734056328844240821172430

**Published:** 2025-02-28

**Authors:** Sebile Çomçalı, Çiğdem Coşkun, Cemal Çavdarlı, Mehmet Numan Alp

**Affiliations:** 1 Department of Ophthalmology, Ankara Bilkent City Hospital, Ankara,Turkey

**Keywords:** Axitinib, Retinal hemorrhages, Cotton-wool spots, Multimodal imaging, Macula edema

## Abstract

**Background:**

Axitinib is a selective inhibitor of vascular endothelial growth factor receptors and is used in the treatment of many malignancies. Herein, we reported a rare case with axitinib-induced retinal changesand associated toxicity.

**Case Presentation:**

A forty-five-year-old female presented with blurred vision who had been taking 7 mg of Axitinib bid for 5 months. Initial Best Corrected Visual Acuity (BCVA) was 20/32 at the right and counting fingers at the left eye. Funduscopic examination revealed bilaterally widespread intraretinal hemorrhages, cotton-wool spots, and hard exudates with a star-like appearance at the macula. The optical coherence tomography revealed central macular edema. There was hyperreflective edema in the inner layers, exudates in the middle retinal layers, and subfoveal subretinal fluid. Fundus fluorescein angiography revealed localized ischaemic findings in the early phase and multifocal perivascular ink-blot fluorescein leakage in the middle and late phases. Axitinib treatment was discontinued immediately, and at the third month of follow-up, the macular edema and fundus findings improved with a final BCVA of 20/20 at the right and 20/32 at the left eye.

**Conclusion:**

Considering the ocular side effects of the patients receiving axitinib is crucial to prevent any potentially persistent visual loss.

## 1 INTRODUCTION

Axitinib is a powerful and selective inhibitor of Vascular Endothelial Growth Factor Receptors (VEGFR) 1, 2, and 3, with proven efficacy in the second-line treatment of renal cell carcinoma [1, 2]. The Phase 3 AXIS study demonstrated its effectiveness in the second-line treatment of advanced Renal Cell Carcinoma (RCC) [3, 4]. It is a promising agent for the treatment of various solid tumors, including thyroid cancer, advanced non-small cell lung cancer, and metastatic melanoma, along with metastatic renal cell carcinoma [5].

The side effects reported in the literature associated with axitinib include tolerable side effects such as diarrhoea, hypertension, fatigue, decreased appetite, nausea, palmar-plantar erythrodysesthesia, weight loss, asthenia, hypo-thyroidism, and rash [6-8].

In this case report, we present retinal toxicity in a patient taking axitinib. The principles of the Declaration of Helsinki were adhered to in the study protocol. Informed consent was obtained, declaring that the patient's medical records and ophthalmologic imaging data can be presented and/or published.

## 2 CASE REPORT

A 45-year-old female patient was presented with a complaint of blurred vision in the left eye for 2 months and in the right eye for 15 days. She had no history of hypertension, diabetes mellitus, or ocular diseases. She had a history of surgery for maxillary adenoid carcinoma in 2018. In March 2021, right hilar lymph adenopathy and widespread metastases were detected in the lung parenchyma, and she received six cycles of chemotherapy. However, due to the progression of lung metastases during follow-up, she had been taking 7 mg of axitinib twice daily for the past 5 months.

In the initial ophthalmological examination, the Snellen Best Corrected Visual Acuity (BCVA) was 20/32 in the right eye and at a level of finger counting from 1 meter distance in the left eye. Her colour vision was tested with Ishihara colour test plates, in which she read 12 of 12 in the right eye and 2 of 12 in the left eye, and an afferent pupillary defect was detected in the left eye.

**Fig. (1):**
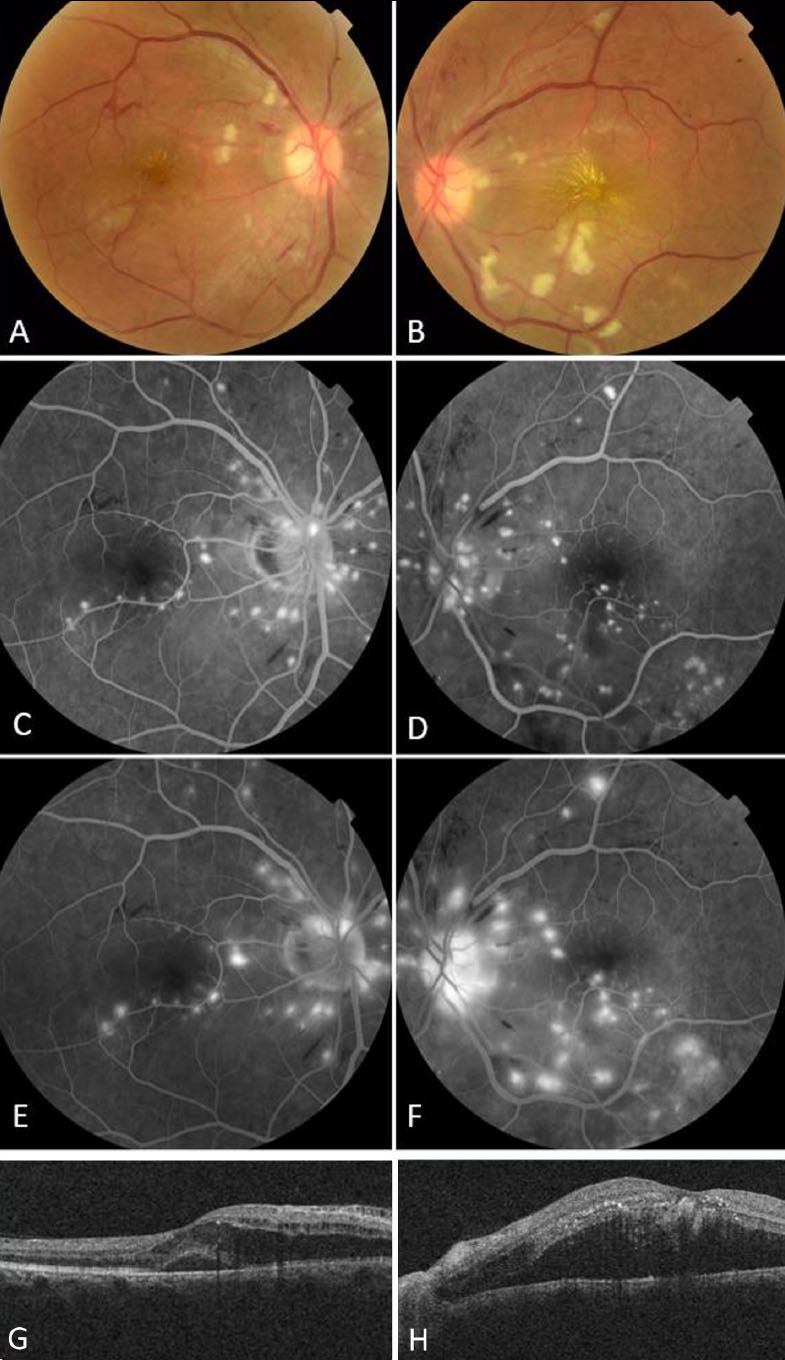
Bilaterally widespread intraretinal haemorrhages, cotton-wool spots, and hard exudates with a star-like appearance in the macula in colour fundus photography. There was a narrowing of the arterioles and dilatation of the veins in both eyes (**A**, **B**). Fundus fluorescein angiography revealed localized ischaemic findings in the early phase and multifocal perivascular (mostly periarteriolar) ink-blot fluorescein leakage in the middle and late phases (**C**, **D**, **E**, **F**, respectively). The optical coherence tomography revealed a central macular thickness of 518μm in the right eye and 965μm in the left eye. There was also hyperreflective edema in the inner layers and subfoveal neurosensory detachment (**G**, **H**).

In the anterior segment examination, the pupil in the left eye was fixed and mid-dilated (4 mm), and other anterior segment findings were normal in both eyes. The right and left intraocular pressures were 14 and 16 mmHg, respectively. Dilated funduscopic examination revealed bilaterally widespread intraretinal haemorrhages, cotton-wool spots, and hard exudates with a star-like appearance in the macula. The left optic disc appeared pale, narrowing the arteriols and dilating the veins in both eyes (Fig. **1A**, **B**).

The Optical Coherence Tomography (OCT) revealed a 1 mm central macular thickness (CMT) of 518μm in the right eye and 965μm in the left eye. There was hyperreflective edema in the inner layers suggestive of ischemia, subfoveal neurosensory detachment, and exudates in the middle retinal layers, and an average increment of the Retinal Nerve Fiber Layer (RNFL) thickness in the initial assessment (167μm and 135μm, respectively) (Fig. **1G**, **H**).

Fundus fluorescein angiography revealed localized ischaemic findings in the early phase and multifocal perivascular (mostly periarteriolar) ink-blot fluorescein leakage in the middle and late phases (Fig. **1C**-**F**).

The patient's blood pressure was within normal limits before and during treatment. Routine blood tests (hemogram, glucose, electrolytes, liver and kidney function, *etc*.) were in a normal range.

The differential diagnosis for bilateral intraretinal haemorrhages, cotton-wool spots, and hard exudates in the macula includes diabetes mellitus, hypertension, retinal vein occlusion, radiation retinopathy, leukemic retinopathy, anaemia, and drug toxicity. In our case, the patient did not have diabetes or hypertension. She did not undergo radiation therapy, and there was no evidence of anaemia.

**Fig. (2):**
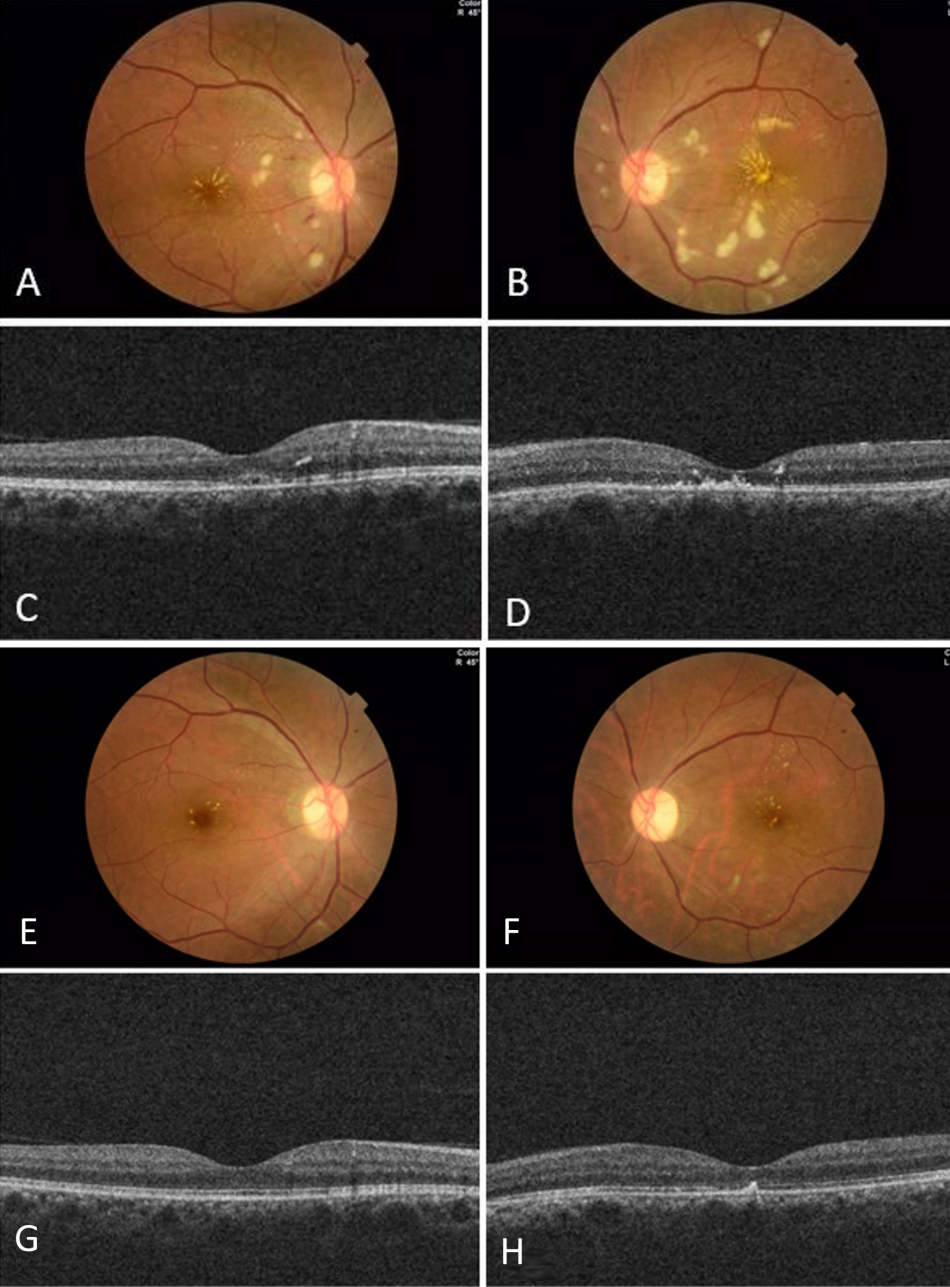
Regression of retinal haemorrhages, cotton-wool spots, and central macular thickness in the third week of follow-up. The left optic disc had a pale appearance (**A**, **B**, **C**, **D**). A distinct improvement in hard exudates in the macula, and a reduction in haemorrhages and cotton-wool spots in colour fundus photography in the third month of follow-up. The optic disc still had a pale appearance (**E**, **F**). The central macular thickness in the optical coherence tomography was 241μm in the right and 209μm in the left eye (**G**, **H**).

The literature on ophthalmic toxicity associated with axitinib is limited to a few reported cases. In the cases reported by Jenkins *et al*., bilateral retinal haemorrhages and cotton-wool spots were present following the use of axitinib, similar to the present case [9]. We consulted with the oncology clinic regarding our patient's suspected ocular toxicity, and as a result, axitinib treatment was discontinued, and everolimus, another tyrosine kinase inhibitor, was initiated. On the fifth day of follow-up, BCVA increased to 6/10 in the right eye and 1/10 in the left eye, with similar ophthalmological findings. On the eleventh day, BCVA increased to 10/10 in the right eye and 1/10 in the left eye, and on the 20th day, it further improved to 10/10 in the right eye and 5/10 in the left eye, with regression of retinal hemorrhages, cotton-wool spots and CMT (Fig. **2A**-**D**). At the 3-month follow-up, BCVA was 10/10 in the right eye and 7/10 in the left eye, with a decrease in hard exudates in the macula and a significant reduction in haemorrhages and cotton-wool spots (Fig. **2E**-**H**). The mid-dilated status of the pupil remained with a slight improvement (3.5mm), and the optic disc still had a pale appearance in funduscopy. During follow-up, a slight improvement on a color vision test was detected in the patient's left eye (5/12), and the relative afferent pupillary defect disappeared.

In control OCT imaging after 3 months of follow-up, the CMT in the right was 241μm and 209μm in the left eye. In addition, the average RNFL thickness improved to normal limits in the right (87μm) and was decreased and atrophic (64μm) in the left eye.

## 3 DISCUSSION

In patients receiving axitinib treatment, a reported incidence of hypertension ranges from 45% to 51% due to increased vascular tone and peripheral resistance [10]. Central retinal vein occlusion has been reported with other oral kinase inhibitors, including sorafenib and regorafenib [11, 12]. The Food and Drug Administration packaging for the medication notes a 1% rate of RVO in clinical trials [13]. In one case report, a patient diagnosed with RCC who had been using axitinib for 2 years presented with widespread cotton-wool spots and significantly delayed filling on FFA [14]. Pyare *et al*. reported a case of a 65-year-old patient with renal cell cancer and subsequent brain metastasis who developed bilateral central retinal vein occlusion and macular edema while receiving 10 mg of axitinib treatment [15].

## CONCLUSION

In conclusion, we presented a case of a patient who developed symptomatic bilateral retinal haemorrhages, cotton-wool spots, and star-shaped exudates in the macula, along with macular edema, following the use of axitinib. The differential diagnosis of these findings observed at the fundus includes diabetes, hypertension, retinal vein occlusion, radiation retinopathy, leukemic retinopathy, and anemia, which were not present in the current subject. The macular edema and fundus findings improved within a short period of time after discontinuation of the axitinib. These side effects tended to resolve shortly after cessation of the drug. Finally, it is crucial to take into account the ocular side effects of axitinib in the routine assessments of the patients receiving this drug.

## Data Availability

The authors confirm that the data supporting the findings of this study are available within the manuscript.

## LIST OF ABBREVIATIONS

VEGFR: Vascular Endothelial Growth Factor Receptors
RCC: Renal Cell Carcinoma
BCVA: Best Corrected Visual AcuityOCT
OCT: Optical Coherence Tomography
CMT: Central Macular Thickness
RNFL: Retinal Nerve Fiber Layer

## STANDARDS OF REPORTING

CARE guidelines were followed.
